# The effectiveness of implementation strategies for promoting evidence informed interventions in allied healthcare: a systematic review

**DOI:** 10.1186/s12913-021-06190-0

**Published:** 2021-03-18

**Authors:** Kaat Goorts, Janine Dizon, Steve Milanese

**Affiliations:** 1 Department of Public Health and Primary Care, Environment and Health, KU Leuven, Leuven, Belgium; 2 International Centre for Allied Health Evidence, University of South Australia, City East Campus, North Terrace, Adelaide, Australia

**Keywords:** Implementation strategy, allied health, health care, evidence based, grades for recommendation

## Abstract

**Background:**

Evidence based practice in health care has become increasingly popular over the last decades. Many guidelines have been developed to improve evidence informed decision making in health care organisations, however it is often overlooked that the actual implementation strategies for these guidelines are as important as the guidelines themselves. The effectiveness of these strategies is rarely ever tested specifically for the allied health therapy group.

**Methods:**

Cochrane, Medline, Embase and Scopus databases were searched from 2000 to October 2019. Level I and II studies were included if an evidence informed implementation strategy was tested in allied health personnel.

The SIGN method was used to evaluate risk of bias. The evidence was synthesised using a narrative synthesis. The National Health and Medical Research Council (NHMRC) model was applied to evaluate the grade for recommendation.

**Results:**

A total of 490 unique articles were identified, with 6 primary studies meeting the inclusion criteria. Three different implementation strategies and three multi-faceted components strategies were described. We found moderate evidence for educational meetings, local opinion leaders and patient mediated interventions. We found stronger evidence for multi-faceted components strategies.

**Conclusion:**

Few studies describe the effectiveness of implementation strategies for allied healthcare, but evidence was found for multi-faceted components for implementing research in an allied health therapy group population. When considering implementation of evidence informed interventions in allied health a multi-pronged approach appears to be more successful.

**Supplementary Information:**

The online version contains supplementary material available at 10.1186/s12913-021-06190-0.

## Background

Evidence-based health care practices have been promoted within healthcare systems internationally [1], as the use of evidence informed practice has been linked to improved patient health outcomes [2]. Clinical guidelines, developed from the best available evidence aim to improve the patient outcomes, quality of care, reduce practice variation and/or reduce cost by providing clinicians with recommendations that reflect best practice [3].

However, the practices recommended in guidelines are not always implemented in healthcare delivery, and significant variations in health care practice remain [1]. It has been suggested that the extent to which guideline implementation occurs depends primarily on two factors: the quality of the evidence on which the guideline is based, and the guideline implementation strategy used [3].

In general, there are two types of implementation strategies; passive strategies, which include the use of educational materials, posters, toolkits and visual aids, or active strategies, which include interactive workshops, academic detailing, audit and feedback and reminders [4]. The evidence suggests that passive strategies may have modest beneficial effects, but do not necessarily lead to sustained behaviour change. In contrast, active multifaceted strategies appear to have the greatest impact [5]. In addition to the type of strategy used, both the individual practitioner and the organization perspectives should be considered in the implementation strategy.

Some authors have suggested that the differentiation between active and passive or single versus multi implementation strategy is too simplistic and fails to recognize the complexity that is inherent in knowledge translation. They advocate for translational strategies that take account of the type of knowledge to be implemented, the context of implementation and the people and processes involved [6]. The PARHIS (Promoting Action on Research Implementation in Health Services) framework [7] described successful translation as a function of the interplay between the research evidence, the context in which translation is happening and the ways in which the process is facilitated. Having one or more people in a facilitatory role, contextualising the evidence and devising appropriate translation strategies for the local environment, forms an important ‘active ingredient’ to the framework.

The Cochrane EPOC group (Effective Practice and Organisation of Care Review Group) has presented a data collection checklist for scientists undertaking reviews into interventions for improving professional practice and the delivery of effective health services. The aim of the checklist is to provide reviewers with guidance on the relevant information that could be extracted from primary studies. This checklist provides an overview of ten (10) different implementation strategies, including both passive and active strategies [8].

The Expert Recommendations for Implementing Change study (ERIC) clustered 73 implementation strategies identified from an expert panel of stakeholders [9] into nine clusters to make it easier to consider the implementation strategies by thematic cluster [10]. These clusters include engaging consumers, using evaluative and iterative strategies, changing infrastructure, adapting and tailoring the context, developing stakeholder interrelationships, utilising financial strategies, supporting clinicians, providing interactive assistance and training and educating stakeholders.

Several studies have investigated the effectiveness of one or multiple implementation strategies, and several systematic reviews have aimed to synthesise this evidence [11–14]. However, in many studies/reviews, the results were not differentiated for the range of professions within the healthcare system, with a number of studies generalizing results for all “healthcare workers” including physicians, nurses, paramedics and other allied health groups. Differentiating between medicine (physicians, doctors), nursing and allied health may be important when considering implementation strategies as adherence to these strategies may differ between these groups.

Three reviews [3, 15, 16] have focused on the allied health profession. However, whilst these reviews gave an overview of the existing evidence, the inclusion of lower quality studies and significant heterogeneity across the included studies meant that the pooling of results was not possible. Also, the recommendations from the evidence on the strategies in practice where not quantitatively graded using grading methods [3, 16]. These inconsistencies may explain the differences in review findings. Menon et al [15], concluded that the use of active, multi-component knowledge transfer interventions enhanced knowledge and practice behaviours in physical therapists but that additional research was needed in occupational therapy. In contrast, Hakkennes and Dodd [3] suggested that multi-faceted interventions were not more effective than single intervention strategies in allied health.

As all three reviews are at least seven years old, it is necessary to update the reviews in light of more current evidence and to explore the recommendations in terms of the quality of the evidence presented and using standardised evidence to decision framework. Therefore, the current review aimed to update the previous evidence reviews by identifying studies that have evaluated the effectiveness of strategies for disseminating and implementing evidence-based guidelines, specifically in an allied health context. By narrowing the review question to this specific context and focussing on high hierarchy and high-quality evidence, we aim to provide more valid recommendations for practice.

## Methods

### Protocol and registration

The systematic review protocol was registered in PROSPERO with ID number 152512

### Identifying the research question

The primary question of this review was to review the effectiveness of implementation strategies for promoting evidence-informed interventions in allied health. A secondary aim was to describe the context in which certain implementation strategies were most effective.’

### Eligibility criteria

Studies were selected based on the study design, the participants, implementation strategies and outcomes. Only randomized controlled trials (RCTs) and systematic reviews (SRs) were included. Within the SRs, only the primary RCTs were included that would satisfy the inclusion criteria.

Data was included if the participants were part of an allied health therapy group. The classification of allied health was based on the definition of Turnbull et. al [17] where four allied health groups were defined: a therapy group, a diagnostic and technical group, a scientific group and a complementary services group. In this paper, we will discuss the allied health therapy group only which includes nutritionist and dietitian, occupational therapist, physiotherapist, psychologist, podiatrist, social worker, speech pathologist, exercise physiologist, ambulance paramedic, music therapist, art therapist, exercise physiologist, ambulance officer, intensive care paramedics).

Studies were included if the implementation strategy was applied to the therapists in the allied health care therapy group (no patient only interventions) and if the implementation strategy was used to implement evidence informed healthcare guidelines. Studies were included if the outcomes addressed the impact on patient outcomes or process/profession outcomes. Studies were excluded if they were not original publications or were not published in the English language or were unable to be accessed in full text.

### Information sources

Keywords were applied in Cochrane, Medline, Embase and Scopus databases on October 4^th^ 2019

### Search

A systematic search was performed to identify literature regarding the effectiveness of research implementation strategies in allied health contexts. The keywords used were: (health* or hospital*).

Allied Health Personnel/ (“allied health personnel” or “allied health professional*” or “assistant*, healthcare” or “health personnel, allied” or “health professional*, allied” or “healthcare assistant*” or “healthcare support worker*” or “paramedic*” or “paramedical personnel” or “personnel, allied health” or “personnel, paramedical” or “population program specialist*” or “professional*, allied health” or “program specialist*, population” or “specialist*, population program” or “support worker*, healthcare” or “worker*, healthcare support”).

“Diffusion of Innovation”/ or Evidence-Based Medicine/ or Evidence-Based Practice/ or Information Dissemination/ (“Knowledge translation” or “knowledge transfer” or “knowledge implementation” or “knowledge utili?ation” or “knowledge dissemination” or “knowledge adoption” or “knowledge change*” or “knowledge evaluation” or “knowledge use*” or “knowledge institutionali?ation” or “knowledge communication” or “research translation” or “research transfer” or “research implementation” or “research utili?ation” or “research dissemination” or “research adoption” or “research change*” or “research evaluation” or “research use*” or “research institutionali?ation” or “research communication” or “evidence translation” or “evidence transfer” or “evidence implementation” or “evidence utili?ation” or “evidence dissemination” or “evidence adoption” or “evidence change*” or “evidence evaluation” or “evidence use*” or “evidence institutionali?ation” or “evidence communication” or “Translation of knowledge” or “translation of research” or “translation of evidence” or “transfer of knowledge” or “transfer of research” or “transfer of evidence” or “systematic review evidence” or “implementation strateg*”).

A date limited search (from 2000 onwards) was applied as the contextual related factors (i.e. healthcare systems) have evolved over time. In addition, the use of formalised evidence-based clinical decision making became popular from approximately 1996 when Sackett and colleagues defined evidence-based clinical decision making as a combination of not only research evidence, but also clinical expertise, taking into account the patient’s preferences [18].

Electronic database searches were supplemented by checking the reference list of included articles.

Searches were performed by two authors (KG and JD).

### Study selection

From the initial search, duplicates were removed. Titles and abstracts were screened for eligibility based on the criteria above and full texts of potentially included studies were retrieved and further assessed for eligibility. Only level I and II studies (SRs and RCTs) were included as they represent the highest level of evidence. Studies were selected independently by two authors (KG and JD).

### Data collation, summary and reporting of findings

A purpose-built Microsoft Excel© sheet was used to extract relevant data from the selected studies including the authors, study design, setting, participants, type of implementation strategy and the associated outcomes. Data was extracted by one author (KG)

Findings were categorised using the taxonomy of professional interventions form [8], and the nine clusters of implementation strategies [10]. The taxonomy of professional interventions include:

- Distribution of educational materials—distribution of published or printed recommendations for clinical care, including clinical practice guidelines, audio-visual materials, and electronic publications
- Educational meetings—health care providers who have participated in conferences, lectures, workshops, or traineeships
- Local consensus processes—inclusion of participating providers in discussion to ensure that they agreed that the chosen clinical problem was important and the approach to managing the problem was appropriate
- Educational outreach visits—use of a trained person who met with providers in their practice settings to give information with the intent of changing the provider’s practice
- Local opinion leaders—use of providers nominated by their colleagues as “educationally influential.” The investigators must have explicitly stated that their colleagues identified the opinion leaders
- Patient mediated interventions—new clinical information (not previously available) collected directly from patients and given to the provider, e.g., depression scores from an instrument
- Audit and feedback—any summary of clinical performance of health care over a specified period of time
- Reminders—patient or encounter-specific information, provided verbally, on paper or on a computer screen that is designed or intended to prompt a health professional to recall information
- Marketing—use of personal interviewing, group discussion (“focus groups”), or a survey of targeted providers to identify barriers to change and subsequent design of an intervention that addresses identified barriers
- Mass media—(i) varied use of communication that reached great numbers of people including television, radio, newspapers, posters, leaflets, and booklets, alone or in conjunction with other interventions; and (ii) targeted at the population level

### Risk of bias in individual studies

Two reviewers (KG and JD) independently assessed the quality of included publications using a relevant critical appraisal tool from the Scottish Intercollegiate Guidelines Network (SIGN) stable [19]. The relevant SIGN checklist was applied to the study and scored with scores < 3 categorised as low quality (LQ), between 4 and 6 average quality (AQ) and > 7 as high quality (HQ). Any disagreements were resolved by discussion between reviewers, and where agreement could not be reached an independent third reviewer (SM) was consulted. The SIGN checklists were used as they are widely used critical appraisal tools that are available for a range of study designs [20].

#### Grading of recommendations

Studies were assessed for relevancy, reliability, validity, and applicability and the level of Evidence was evaluated using the National Health and Medical Research Council (NHMRC) model for additional levels of evidence and grades for recommendations for developers of guidelines. The NHMRC model is a logical and intuitive way to formulate and grade recommendations that has been widely adopted by Australian guideline developers [21]. The grading process of the NHMRC process is described in Table 1 of the supplementary files.

## Results

### Study selection

**Fig. 1:**
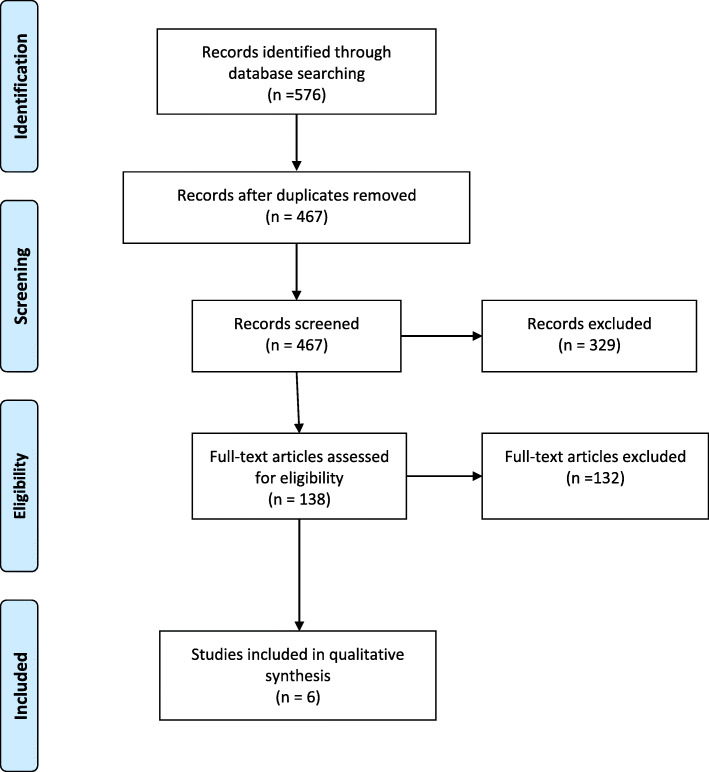
PRISMA flow chart

The initial search yielded 464 original results, however only six studies remained for inclusion after screening (see Fig. 1). We found two eligible RCTs and two eligible SRs. From one SR [3], no overview table was available, and it was therefore decided to screen the reference list from this review to find eligible studies. Since all eligible primary studies from this review [3] were also included in the second review [16], it was decided to exclude this review.

We decided to include the primary studies from the SR. A total of six studies were included (two primary studies from the search and four primary studies from the SR. [16]

### Study characteristics

Studies were grouped and categorised by implementation strategy based on the EPOC Taxonomy and the ERIC clusters. The results from the individual studies are summarized in Table 1.

**Table 1:** Description of individual studies

Author Year Country	Setting/Allied health	Strategies		Outcomes	Findings	SIGN SCORE
		Implementation intervention/strategy (Cluster)	Control			
Stevenson et al 2006 [23] (UK)	Community Trust physiotherapy AH: physiotherapists	**Opinion leader** Educational program administered by local opinion leaders 5 hours	**‘Usual’ in-service training.** A standard in-service training package on clinical management of knee dysfunction and pathology	**Patient classification** Classification in three categories: acute low back pain; subacute low back pain, or chronic low back pain.	• **Clinical management: relatively unchanged**	AQ+
		**(Training and educating stakeholders)**	5 hours	**Time spent** Rank management approached regarding time spent **Importance** Rank management approaches regarding importance		
Snooks 2014 (UK) [24]	Ambulance stations AH: Paramedics	**Patient mediated intervention** CCDS (Computerised Clinical Decision Support) on hand-held Tablet computers to decide whether to take patients who had fallen to an Emergency Department or leave them at home with referral to a community-based falls service **(Providing interactive assistance)**	**Usual care** Paper based protocols to assess patients and make decisions about their care Care in control group was not standardised.	**Effectiveness** Proportion of participants left at scene without conveyance to an Emergency Department versus proportion referred to falls services **Safety** Proportion of participants with adverse events [20] up to one month (999 call, Emergency Department attendance, emergency admission to hospital, or death) **Cost-effectiveness** Costs of implementation of CCDS for paramedics and its benefits in the form of patient utility modelled over 12 months **Self reported falls** Fall-related self efficacy **Health related quality of life** SF12 **Patients satisfaction** Quality of care monitor	• 17 intervention paramedics used CCDS for 54 (12.4%) of 436 participants. • 9.6% referred to falls services versus 5.0% in the control group • Odds ratio (OR) 2.04, 95% CI 1.12 to 3.72. • **No adverse events were related to the intervention** • **CCDS is potentially cost-effective, especially with existing electronic data capture.**	AQ+
Bekkering 2005 (a) [25] (The Netherlands)	Physiotherapy practices AH: physiotherapists	**Educational outreach visit** **Audit and feedback** **Reminders** Two training sessions 2.5 hours (each) Supervised by primary investigator and one of two additional trainers with adequate clinical experience in the management of low back pain **(Training and educating stakeholders + using evaluative and iterative strategies)**	**Standard passive method of dissemination** Guidelines are send by mail, along with 4 forms to facilitate use	**Adherence to the guidelines** Individual patients’ forms recording the treatment completed by the physiotherapist. Forms were assessed using an algorithm based on the number of treatment sessions, treatment goals, interventions, and patient education	• Correctly limited the number of treatment sessions for patients with a normal course of back pain (OR 2.39; 95% CI 1.12 to 5.12) • Set functional treatment goals (OR 1.99; 95% CI 1.06 to 3.72) • Used mainly active interventions (OR 2.79; 95% CI 1.19 to 6.55), • Gave adequate patient education (OR 3.59; 95% CI 1.35 to 9.55). • Adhered more to all four criteria (OR 2.05; 95% CI 1.15 to 3.65). • **The active strategy moderately improved adherence to the guidelines.**	HQ++
Bekkering 2005 (b) [26] (The Netherlands)	Physiotherapy practices AH: Physiotherapists	**Educational outreach visit** **Audit and feedback** **Reminders** Two training sessions 2.5 hours (each) Supervised by primary investigator and one of two additional trainers with adequate clinical experience in the management of low back pain **(Training and educating stakeholders + using evaluative and iterative strategies)**	Standard passive method of dissemination Guidelines send by mail, along with 4 forms to facilitate use	**Patient outcomes** Self-report questionnaires at baseline and 6, 12, 26, and 52 weeks after baseline **Physical functioning** (QBPDS),19,20 **Pain** (11-point numeric rating scale [NRS]),22,23 **Sick leave** Number of days off work in the last 6 weeks	• Physical functioning: 2.83 points difference on QBPDS (95% CI: -.66, 6.31) • Pain: 0.34 points difference on NRS ((95% CI: -.19, .88) • Sick leave: no results (only 7% on sick leave at 12 months) • **No additional benefit to applying an active strategy to implement the physical therapy guidelines for patients with low back pain.**	HQ++
Pennington et al 2005 [22] (UK)	Management of post stroke dysphagia AH: Speech language therapists	**Educational meetings** Five days training once per fortnight at Manchester University from April to June 2002. Same as control group with 2,5 days of additional training on the diffusion of innovation, using the model developed by Rogers. **(Training and educating stakeholders)**	2,5 days training over seven weeks (April to May 2002) Manchester University. Introduction to clinical governance and evidence-based health care, critical appraisal of systematic reviews, randomized controlled trials, cohort and quasi experimental studies and evidence-based guidelines	**Adherence to practice guidelines** Using a process-based audit tool, developed by the researchers and a consensus group **Cost effectiveness** 3 categories of costs: providing the two training strategies, attending the two training strategies and rolling out the training to the rest of the SLT department	• No significant effect on initial compliance (F=0.16, df 1, 15, p=0.9) • No significant overall response to training (F= 1.33, df 1, 1436, p 0.25) • No effect of training strategy on post-intervention compliance (F 2.80, df 1, 15, p =0.12) • Departments' rating of research culture included in model improved the significance of the effect of strategy on response to training (F 3.66, df 1, 11, p 0.08) • Increased dissemination activities and awareness of research information • **No changes in clinical practice within six months of training.** • Costs of the roll out of training for both strategies • No relationship between costs and clinical outcome.	HQ++
Rebbeck 2006 [27] (Australia)	Physiotherapy clinics AH: physiotherapists	**Distribution of educational materials** **Opinion leaders** **Follow-up education** **Educational meetings (workshops)** **Educational outreach visits** 8 h workshop including interactive sessions outlining the content of the guidelines, practical sessions covering the treatments endorsed in the guidelines Local opinion leaders delivered some of the program content. Algorithms outlining the process of care, appointment cards, and marketing material to be used for general practitioners who usually refer to the practice Follow-up educational outreach visit (2 hours) 6 months later: problem solving regarding use of the guidelines in clinical practice and update of the evidence **(Training and educating stakeholders + adapting and tailoring the context + supporting clinicians)**	**Dissemination of guidelines** By mail Physiotherapists were given but not directed to use the guidelines. Both groups were given the same information regarding the trial and its outcome measures	**Patient outcomes: disability, disability due to acute whiplash, whiplash, clinically important change, patient satisfaction** Functional Rating Index, adapted version of the 7-item Core Outcome Measure for neck pain, 5item questionnaire ‘symptom bothersomeness’, Global Perceived Effect, 5-point Likert scale ranging from 1 (extremely dissatisfied) to 5 (extremely satisfied) **Physiotherapist outcomes: knowledge, clinical practice, physiotherapists satisfaction** custom-made questionnaire, percentage prescribing guideline recommendations before and after the trial (from responses to the questionnaire) and during the trial (audited from patient notes), 7-point Likert scale ranging from –3 (extremely unhelpful) to +3 (extremely helpful) **Cost of care** Median cost per patient for each physiotherapist.	• No significant difference for any of the patient outcomes • Increased their knowledge of the guidelines by 5.5 points (95% CI 2.5 to 8.4) (p = 0.001) • Increased self-rated understanding of the guidelines by 1.5 points (95% CI 0.7 to 2.3) (p = 0.001). • Increased ability to identify yellow flags (p = 0.02) • Increased self-reported use of functional outcome measures (p = 0.01) • 2/5 guideline recommendations were identified by more ‘reassure patient’ (p = 0.05) and ‘advise to act as usual’ (p = 0.02). • Recommendations prescribed more (p = 0.04 and 0.02) • Equal satisfaction with the guidelines (p = 0.29) or the consumer version of the guidelines (p = 0.20) • More satisfied with implementation package (p = 0.07) • Cost of care not significantly different (p = 0.67) • Cost per one point improvement not significantly different (p = 0.55) • Median of 13 treatments to patients in the implementation group not significantly different (p = 0.75) • **Improved knowledge and clinical practice more consistent with the guidelines** • **Patient outcomes and cost of care were not affected.**	HQ++

Three types of implementation strategies were identified. One study described educational meetings [22], one study described local opinion leaders [23], one study described patient mediated intervention [16] and three studies described multi-faceted components [24–26]. Four studies involved physiotherapists [23, 25, 26, 27], one with paramedics [24] and one with speech language therapists [22].

Three studies were from the UK [22–24], two from the Netherlands [25, 26] and one from Australia [27].

### Outcomes

The outcomes for each implementation strategy are summarized in Table 2.

**Table 2:** Synthesis of results

Interventions (EPOC strategies)	Study	Allied health	Outcomes	
			Patient/ health	Professional/Process
Educational meetings	Pennington et al 2005 [22]	Speech-language therapists	• Not evaluted	• Pre- and post-training adherence to practice guidelines (increased dissemination activities (S) • Knowledge increased (S) • Changes in clinical practice within six months of training (NS) • Cost effectiveness (NS)
Local opinion leaders	Stevenson et al 2006 [23]	Physiotherapists	• Not evaluted	• Change in physio practice (NS) • Patient classification (NS) • Time spent (NS) • Importance (NS)
Patient mediated interventions	Snooks 2014 [24]	Paramedics	• Patient safety (NS) • Self-reported falls (NS) • Health related quality of life (NS) • Patient satisfaction (NS)	• Cost effectiveness (S) • Increased patient referral to falls services (S)
Multi-faceted components	Rebbeck 2006 [27] • Opinion leaders • Follow-up education. • Educational meetings (workshops) • Educational outreach visits	Physiotherapists	• Patient disability (NS) • Patient satisfaction (NS)	• Knowledge (S) • Guideline adherence (S) • Cost effectiveness (NS)
	Bekkering 2005 (a) [25] • Educational outreach visit • Audit and feedback • Reminders	Physiotherapists	• Not evaluated	• Guideline adherence (S)
	Bekkering 2005 (b) [26] • Educational outreach visit • Audit and feedback • Reminders	Physiotherapists	• Physical functioning, pain and sick leave (NS)	• Not evaluated

S = significant (*p*<0.05) NS= Not significant (*p*>0.05)

The grades of recommendation according to the NHMRC model are listed in Table 3.

**Table 3:** Grades of recommendation

Strategies	Grade of Recommendation (NHMRC)
**●** Educational meetings improve therapists’ knowledge and adherence to guidelines but have no effect on clinical practice, patient-related outcomes or cost effectiveness.	B
**●** Local opinion leaders have no effect on professional or process outcomes	C
**●** Patient mediated interventions are cost effective and increase patient referral to falls services but have no effect on patient outcomes (patient safety, self-reported falls, health-related quality of life and patient satisfaction).	C
**●** Multi-faceted interventions improve therapists’ knowledge and adherence to guidelines but have no effect on clinical practice, patient-related outcomes or cost effectiveness.	A

Whilst educational meetings were found to have a significant positive effect on therapists’ adherence to guidelines and knowledge increase, no patient-related outcomes were measured, and no significant changes were reported in clinical practice or cost effectiveness. The overall NHMRC grade of recommendation was B, suggesting that the recommendation can be trusted to guide practice in most situations.

We found no significant effect of local opinion leaders on professional or process outcomes, however no patient outcomes were explored for this strategy. The overall NHMRC grade of recommendation was C, suggesting that the body of evidence provided some support for the recommendation(s) but care should be taken in its application.

For patient mediated interventions, the review found significant effects on cost effectiveness and a significant increase in patient referral to falls services. However, all patient outcomes (patient safety, self-reported falls, health-related quality of life and patient satisfaction) did not significantly differ from the control group. The overall NHMRC grade of recommendation was C.

The body of evidence related to multi-faceted intervention strategies provided the highest grade of recommendation (A), suggesting that this recommendation can be trusted to guide practice. The review found that multi-faceted component studies improved guideline adherence significantly in two studies [25, 27] and knowledge in one study [27].

When considering the evidence implementation interventions in terms of clusters the most common cluster of implementation strategy utilised involved training and educating stakeholders, when used in isolation this implementation strategy cluster was the least effective. When interventions were used that spanned a range of clusters the effectiveness of the implementation strategies appeared stronger. The strongest evidence of effectiveness came from the implementation of interventions that spanned the clusters of training and educating stakeholders, adapting and tailoring the context and supporting clinicians [27].

### Risk of bias within studies

The six studies included in this review were of sound methodologic quality with SIGN scores ranging from adequate or high quality (AQ or HQ) [19]. (see additional files). All studies had a clear purpose, relevant background, and justification for conducting the study. Randomization was not clearly described in one study. In two studies, treatment and control group were not described at the start of the trial and no adequate concealment method was applied. All studies had adequate blinding and the only difference between groups was treatment under investigation. All studies but one described the dropout rate. Intention to treat analysis was executed for only three studies.

### Summary of changes from the study protocol

During the review process the following items were changed from the study protocol. Due to the nature of the evidence found we decided to include only level I (systematic reviews) and II (RCT) studies in this paper. Only quantitative studies were considered, and implementation strategies were specified using the EPOC framework. The SIGN checklist and NHMRC grading framework was used to categorise the risk of bias and synthesize the results respectively.

## Discussion

This is the most recent review exploring the effectiveness of implementation strategies in allied healthcare. Six studies related to allied health were found but only among physiotherapists, speech pathologists and paramedics. Strategies evaluated were educational meetings, use of local opinion leaders, patient mediated interventions and a combination of different strategies forming multi-faceted interventions. Most strategies were evaluated against professional and process outcomes and only half were evaluated against patient or health outcomes. Multi-faceted strategies appear to remain the most effective in improving knowledge and adherence to guidelines and evidence (professional outcomes) but none of the strategies were found to improve patient outcomes.

Despite over 20 years since the recognition of the importance of evidence-based practice in quality health care this review could only identify six studies that explored the effectiveness of implementation strategies for promoting evidence-informed interventions in allied health. It was important to limit to the search to allied health as profession -related health discipline practice differences make it unlikely that the evidence associated with medicine would automatically transfer across to allied health. Whilst there has been an exponential growth in published evidence-based research across all allied health disciplines this has not been matched by published research into how best to implement this in clinical practice. Without effective strategies for implementation of evidence-based recommendations it is unlikely that evidence-based practice will improve the quality of care, reduce practice variation and/or reduce cost.

The importance of the implementation strategy to the effective use of evidence-based practice has been recognised by numerous authors [7, 8]. Without a good understanding of the most effective strategy for implementing evidence-based recommendations in the real-world evidence-based practice becomes purely an academic exercise. Ecological validity depends on the evidence-based practice recommendation being tested in the real world. The current body of evidence related to implementation strategies in allied health are limited to speech-language therapists, paramedics and physiotherapists.

Of the evidence that exists there is relatively stronger support for the use of intervention strategies that are multi-faceted, including a range of active and passive strategies, rather than uni-faceted strategies such as educational meetings, local opinion leaders and patient mediated interventions. This adds support to the findings of Menon et al [15], who found multi-component knowledge transfer interventions enhanced knowledge and practice behaviours in physiotherapists. This is particularly evident when the interventions are explored in terms of clustering with the strongest evidence of effectiveness coming from strategies that include interventions from a range of clusters [27].

Across the studies found in this review there was inconsistent outcomes explored. Guideline adherence and knowledge were the two most common outcomes that were measured, potentially reflecting the relative ease of data capture of these two measures. Of concern when considering the body of evidence is the lack of focus on patient-centred outcomes. If the aim of evidence-based recommendations is to improve the health care of patients then this should be reflected in the evidence associated with intervention strategies. Patient-reported outcome measures would appear to be an important indicator of the effectiveness of an intervention strategy in improving patient centred care.

Whilst multifaceted interventions demonstrated the greatest effect on improving guideline adherence and knowledge the lack of changes in clinical patient outcomes is a concern. It is difficult to demonstrate cost effectiveness of an intervention if there are not measurable changes in patient outcomes. There remains limited evidence, from the findings of this review, that interventions based on training and educating stakeholders, adapting and tailoring the context and supporting clinicians change patient outcomes. This suggests either that the patient-related outcome measures were not sensitive enough or that different intervention strategies are needed to change patient outcomes. More research is needed in this area.

Due to the strict inclusion criteria of including only allied health therapy disciplines, only a few studies were found. Whilst this may be perceived as a limitation of the current review it also ensures that the reviews findings are relevant to the allied health discipline and reinforce the continued limited evidence base available in evaluating implementation strategies in allied health. This review is also limited by its focus on publications in the English language only.

## Conclusions

The current limited evidence base in allied health suggests that multifaceted interventions, including the use of opinion leaders, follow-up education, educational meetings (workshops), audits and feedback and reminders, appear to be the most effective in implementing evidence-based recommendations. Therefore, when considering the use of evidence informed interventions in allied health an implementation strategy that incorporates these should be developed. Whilst evidence for knowledge uptake and guideline adherence and increased referrals exist there remains little consideration for patient or health related outcomes.

## Supplementary Information

**Additional file 1 Table S1.** Critical appraisal scores.

## Data Availability

Data sharing is not applicable to this article as no datasets were generated or analysed during the current study.

## Abbreviations

SIGN: Scottish Intercollegiate Guidelines Network
NHMRC: National Health and Medical Research Council
PARHIS: Promoting Action on Research Implementation in Health Services
EPOC: Effective Practice and Organisation of Care Review
PROSPERO: International prospective register of systematic reviews
ID: Identification
RCT: Randomized controlled trials
SR: Systematic reviews
LQ: Low Quality
AQ: Average Quality
HQ: High Quality
