# Supplementary material for: The effectiveness of implementation strategies for promoting evidence informed interventions in allied healthcare: a systematic review
Source: BMC Health Serv Res. 2021 Mar 18;21:241. doi: 10.1186/s12913-021-06190-0 (PMC7977260; doi:10.1186/s12913-021-06190-0)
Supplement: Supplementary file 1 — Additional file 1 Table S1. Critical appraisal scores. [file 12913_2021_6190_MOESM1_ESM.docx]

Table 1: Critical appraisal scores

| Q. | Reference (Author, year) | Stevenson 2006 | Snooks 2014 | Bekkering 2005(a) | Bekkering 2005(b) | Pennington 2005 | Rebbeck 2006 |
| --- | --- | --- | --- | --- | --- | --- | --- |
| 1.1 | The study addresses an appropriate and clearly focused question | Y | Y | Y | Y | Y | Y |
| 1.2 | The assignment of subjects to treatment groups is randomised | Can’t say | Y | Y | Y | Y | Y |
| 1.3 | An adequate concealment method is used | N | N | Y | Y | Y | Y |
| 1.4 | The design keeps subjects and investigators ‘blind’ about treatment allocation | Y ([participant) | Y  (assessor) | Y | Y | Y | Y |
| 1.5 | The treatment and control groups are similar at the start of the trial | Can’t say (not all) | Can’t say | Y | Y | N | Y (Physio)  N (patients) |
| 1.6 | The only difference between groups is the treatment under investigation | Y | Y | Y | Y | Y | Y |
| 1.7 | All relevant outcomes are measured in a standard, valid and reliable way | Y | Can’t say | Can’t say | Y | Y | Y |
| 1.8 | What percentage of the individuals or clusters recruited into each treatment arm of the study dropped out before the study was completed? | Patients  I (18%)  C (13%) | Can’t say | Patients  I (29%)  C (21%) | Patients  I (13%)  C (14%) | Patients  I (7%)  C (3%) | Patients  I (7%)  C (16%) |
| 1.9 | All the subjects are analysed in the groups to which they were randomly allocated (intention to treat analysis) | N | N | Y | Y | Y | N |
| 1.10 | Where the study is carried out at more than one site, results are comparable for all sites | Can’t say | Can’t say | Can’t say | Can’t say | Can’t say | Can’t say |
| 2.1 | How well was the study done to minimise bias? | AQ (+) | AQ (+) | HQ (++) | HQ (++) | HQ (++) | HQ (++) |
| 2.2 | Taking into account clinical considerations, your evaluation of the methodology used, and the power of the study, are you certain that the overall effect is due to the study intervention? | Y | Y | Y | Y | Y | Y |
| 2.3 | Are the results of this study directly applicable to the patient group targeted by this guideline? | Y | Y | Y | Y | Y | Y |
